# Cytotoxic and growth inhibitory effects of the methanol extract **Struchium sparganophora** Ktze (Asteraceae) leaves

**DOI:** 10.4103/0973-1296.71795

**Published:** 2010

**Authors:** B. A. Ayinde, U. Agbakwuru

**Affiliations:** *Department of Pharmacognosy, Faculty of Pharmacy, University of Benin, Benin City, Nigeria*

**Keywords:** Cytotoxicity, growth inhibitory, Struchium sparganophora

## Abstract

**Background:**

Global research into medicinal plants used in treating tumor-related ailments has become imperative due to the emergence of various forms of cancer diseases. Usually consumed as a vegetable, **Struchium sparganophora** is indicated in traditional herbal medicine as one of the plants used in treating tumor-related ailments.

**Materials and Methods:**

This claim was examined using bench-top assay methods involving the cytotoxicity of the methanol extract of the leaves to tadpoles of **Raniceps ranninus** at 10, 20, 40 and 80 *μ*g/ml. Also, the growth inhibitory effects of the extract on guinea corn radicle at 0.5, 1.0, 2 and 4 mg/ml in addition to evaluation of the phytochemical constituents of the leaves was performed. After 24 h, the crude extract and the chloroform fraction produced the highest cytotoxicity of 96.67 ± 4.71%, each at a concentration of 80 *μ*g/ml, while the aqueous fraction produced 100% cytotoxicity at a concentration of 20 *μ*g/ml.

**Results:**

The crude extract had an LC50 of 26 *μ*g/ml, the chloroform fraction had 6.25 while the aqueous fraction had 5 *μ*g/ml. On the inhibition of the guinea corn radicle growth, after 96 h, the controls had an average length of 67.81 ± 2.6 mm, whereas the seeds treated with 4 mg/ml of the crude extract had an average length of 35.83 ±1.75 mm, indicating 47.81% reduction in length. At the same concentration, the chloroform and the aqueous fractions showed 32.51 and 43.81% inhibitions. The plant material was observed to contain alkaloids, tannins, saponins and flavonoids, with no traces of anthracene derivatives.

**Conclusion:**

The results suggest the probable use of the plant in preparing recipes for tumor-related ailments.

## INTRODUCTION

Traditional herbal medicines are getting significant attention in global health discussions. About 80% of the African population relies on herbal medicines to complement its health needs and many countries, including Nigeria, have been reported to make substantial research investments into the practice.[1] In various communities where there is a gross, uneven distribution of orthodox health personnel, cure for many ailments and disease conditions is usually attempted by herbalists using medicinal plants widely distributed in the wild. In such communities, cure for life-threatening disease condition characterized by tumor production is attempted using medicinal plants. In the literature, there a lot of compounds ranging from lignans like 5-methoxypodophyllotoxin[2] to terpenoids like taxol[3,4] that were originally obtained from natural sources but were later developed to full anticancer agents.

In the scientific world, the search for antitumor agents of natural origin continues to increase due to the non-selective pattern in the activities of the conventional drugs coupled with their high costs and scarcity, particularly in rural areas. Research into antitumor agents usually involves a series of complex procedures that sometimes produces non-encouraging results after much material and time has been expended. In addition to these, paucity of research funds has made the development and acceptance of simple bench-top assays imperative.[5]

Commonly called Ewuro odo in Yoruba, **Struchium sparganophora** (Linn.) O. Ktze (Asteraceae) is a vegetable usually used in the preparation of soup in the South Western part of Nigeria. It is a shrub that occurs more commonly as a cultivated species than as a wild plant growing near the waterside.[6] In ethnomedicine, the plant was reportedly useful in the treatment of cutaneous, subcutaneous parasitic infection, diarrhea, dysentery as well as venereal diseases. It is also useful as an abortifacient.[7] Furthermore, it is reportedly useful in the treatment of rheumatic pains.[8] Based on the additional ethnomedicinal application of the plant as one of the medicinal plants used in the treatment of inflammatory and tumor-related ailments, this work was carried out to examine the cytotoxic and antiproliferative effect of the methanol extract of the leaves using bench-top assay methods involving tadpoles of **Raniceps ranninus** and guinea corn *Sorghum bicolor* radicle length.

## MATERIALS AND METHODS

The leaves of *S. sparganophora* were collected in July 2008, in Iwo, Osun State, Nigeria. The identity of the plant was confirmed by Dr. Shasanya Olufemi, the Plant Taxonomist at the Forest Research Institute of Nigeria (FRIN), Ibadan. A herbarium specimen number FHI 108438 was deposited at the institute for reference. The plant material was air dried in the laboratory for 5 days at room temperature followed by oven drying at 40° C followed by grinding to powder form using an electric mill. The powdered sample was kept in an air tight container until required.

### Phytochemical screening of the plant material

This was carried out to test for the presence or otherwise of tannins, flavonoids, saponins, alkaloids, steroidal glycosides and anthracene derivatives using standard methods.[9,10]

### Extraction of the plant material

About 450 g of the powdered leaves of *S. sparganophora* was macerated in 2.5 l of methanol for 72 h. After filtration and concentration using a rotary evaporator maintained at 40° C, the extract obtained was weighed (27.3 g; 6.07%) and kept in the refrigerator.

### Organic solvent partitioning of the extract

About 18 g of the leaf extract of *S. sparganophora* was dissolved in methanol–water (1:1) and exhaustively partitioned with chloroform (3 X 200 ml). The chloroform and aqueous fractions obtained were concentrated using a rotary evaporator. The chloroform fraction was 5.3 g while the aqueous fraction weighed 9.1 g.

### Sources and identification of the tadpoles of **Raniceps ranninus**

Tadpoles were harvested from toad colonies in small water settlements around the Faculty of Pharmacy, University of Benin. They were identified as the tadpoles of **Raniceps ranninus** by Professor M. Aisien, Animal Parasitologist, Department of Animal and Environmental Biology, Faculty of Science, University of Benin. Based on the information provided, 5–6-day-old tadpoles were removed and separated from others.

### Source and preparation of the guinea corn *Sorghum bicolor*

Guinea corn obtained from a local market in Benin was cleansed with absolute alcohol after which the seeds were dried before use. The viability of the seeds was determined by their ability to remain submerged in water. Those that remained submerged in water were selected and dried for use.

### Determination of the cytotoxic effects of the methanol extract and the organic solvent fractions on tadpoles (**Raniceps ranninus**)

Using a modification of a method obtained from the literature,[11] ten (10) tadpoles were selected in 50-ml capacity beakers containing 15 ml of the water from the source of the tadpoles, which was made up to 49 ml with distilled water. The volume was made up to 50 ml with 0.5 mg/ml, 1 mg/ml, 2 mg/ml and 4 mg/ml of the extract dissolved in 5% dimethyl sulfoxide in water, thereby making concentrations of 10, 20, 40 and 80 μg/ml, respectively. The procedure was repeated using the chloroform and aqueous fractions obtained from the partitioning process stated above. For the extract and the organic solvent fractions, the experiment was carried out in triplicates. The controls for each of the experiments were not treated. The mortality rates of the tadpoles were observed for a maximum of 24 h.

### Determination of the growth inhibitory effects of the methanol extract and the organic solvent fractions on guinea corn (*Sorghum bicolor*)

About 10 ml of 0.5 mg/ml, 1 mg/ml, 2 mg/ml and 4 mg/ml of the methanol extract dissolved in 5% dimethyl sulfoxide in water was poured into 9-cm-wide Petri dishes laid with cotton wool and filter paper (Whatman No 1). Twenty (20) viable seeds were spread on each plate and incubated in a dark environment. The lengths (mm) of the radicles emerging from the seeds were taken at 24, 72 and 96 h. The control seeds were treated with 10 ml of 5% dimethyl sulfoxide in distilled water containing no extracts. The experiments were carried out in triplicates. The procedure was repeated for both the chloroform and aqueous fractions obtained from the partitioning process of the methanol extract.

## RESULTS

Preliminary phytochemical examination of the powdered sample of *S. sparganophora* revealed the presence of alkaloids, tannins, saponins and flavanoids. The plant material was observed to be devoid of cyanogenic glycosides and anthracene derivatives [Table 1].

**Table 1:** The results of the preliminary phytochemical constituents of *S. sparganophora* leaf powder

Phytochemical constituents	Results
Tannins	++
Saponins	++
Cynanogenic glycosides	−
Alkaloids	++
Anthracene derivatives	−
Flavonoids	++

### Cytotoxic effects of the extract and the organic solvent fractions

**Figure 1:**
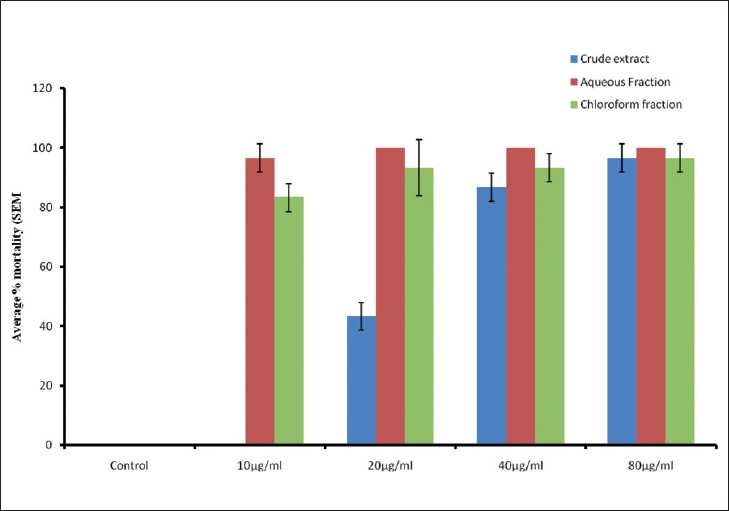
Effects of the extract methanol and organic solvent fractions of *S. sparganophora* on tadpole

At the end of the 24-h period, the methanol extract and the organic solvent fractions of *S. sparganophora* were observed to exhibit remarkable cytotoxicity on the tadpoles. The crude extract produced a mortality of 43.33 ± 4.71% at a concentration of 20 μg/ml, which increased to 96.67 ± 4.71% with 80 μg/ml. The aqueous fraction produced the highest cytotoxic effects as it recorded 96.67 ± 4.71 and 100% mortalities at concentrations of 10 μg/ml and 20 μg/ml, respectively. However, the chloroform fraction showed the highest mortality of 96.67 ± 4.71% at 80 μg/ml. The cytotoxic effects were indicated by an initial slow down in the movements of the tadpoles and subsequent ceasation of movements, indicated by complete submergence and turning upside down of the organisms. No mortality was recorded in the controls [Figure 1]. The effects of the extract and the fractions were observed to be significantly different from the controls (*P* < 0.001). While an LC50 of 24 μg/ml was obtained for the crude extract, LC50s of 5 and 6.25 μg/ml were observed for the aqueous and the chloroform fractions, respectively.

### Effect of the extract on the length of the radicle

The extract was observed to reduce the rate of germination of the seeds with increasing concentrations. The lengths of the seed radicles increased with the incubation period of 24–96 h. While those of the control seeds increased progressively, the methanol extract of the leaves of *S. spaganophorus* was observed to elicit concentration-dependent reductions in the length of the radicles that emerged from the guinea corn seeds treated with the extract and the organic solvent fractions.

**Figure 2:**
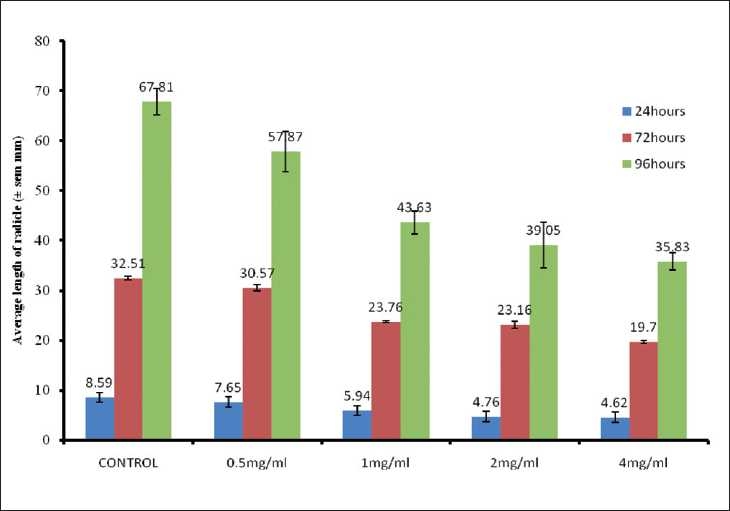
Inhibitory effects of the methanol extract of *S. sparganophora* leaves on length of *Sorghum bicolor* radicle

The average length of the radicles at 24 h was 8.59 ± 0.76 mm in the control seeds, while the average length in the seeds treated with 4 mg/ml of the extract was 4.62 ± 0.39 mm, indicating a 46.22% reduction in the length. After 96 h, the control seeds had an average length of 67.81 ± 2.6 mm, whereas the seeds treated with 2 and 4 mg/ml of the extract showed average lengths of 39.05 ± 4.6 and 35.83 ±1.75 mm, also indicating 42.41 and 47.81% reductions, respectively [Figure 2].

**Figure 3:**
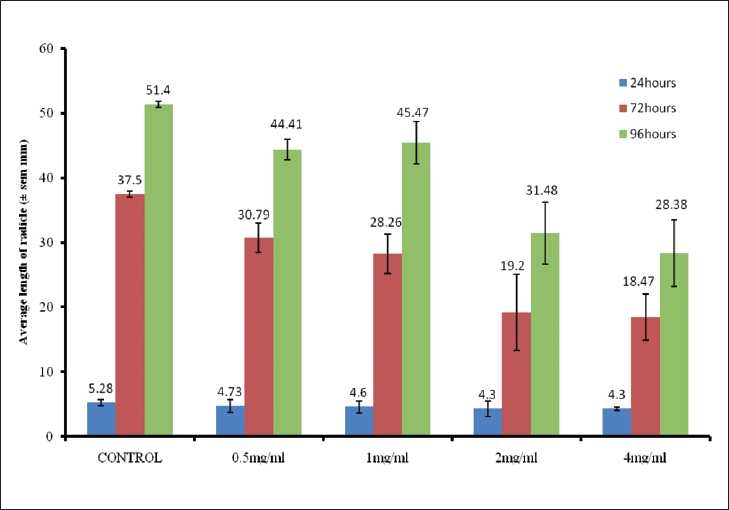
Inhibitory effects of the aqueous fraction of *S. sparganophora* leaves on length of *Sorghum bicolor* radicle

**Figure 4:**
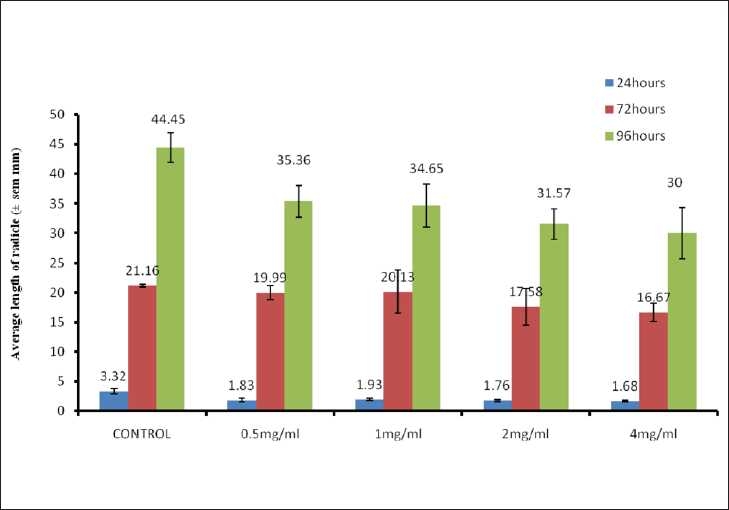
Inhibitory effects of the chloroform fraction of *S. sparganophora* leaves on length of *Sorghum bicolor* radicle

The various concentrations of the aqueous and chloroform fractions were equally observed to inhibit the growth of the radicles of the seeds. The aqueous fraction was observed to be more effective than the chloroform fraction. At 96 h, the average length of 51.4 ± 2.25 mm in the controls was reduced to 31.48 ± 1.89 mm and 28.88 ±2.09 mm in seeds treated with 2 and 4 mg/ml concentrations, showing 38.76 and 43.81%, reductions respectively [Figure 3]. The chloroform fraction reduced the control lengths of 44.45 ± 2.67 to 31.57 ± 2.55 and 30.0 ± 4.3 mm, indicating 29 and 32.51% reductions, respectively [Figure 4].

## DISCUSSION

Production of tumor cells is characterized by an uncontrolled multiplication of the cells. This can be linked to the rapid growth and multiplication exhibited by the meristematic cells of a germinating seed or a growing radicle.

Bench-top assay methods have been variously used to investigate the ability of plant extracts to impart cytotoxicity on certain zoological organisms like the nauplii of *Artemisia salina*[5] mosquito larvae and tadpoles.[11] Apart from the reproducibility the methods provide, they are simple and can be used to screen medicinal plants with probable antitumor effects. The use of the organisms mentioned above and also mosquito larvae has been reported to be a measure of the plant extract to inhibit growth of tumor-producing cells, induce dormancy in plant seeds or produce allelopathic effects.[5] The tadpoles were used in this work due to their availability, particularly in the raining season. This of course could be a limiting factor in carrying out this kind of work in the drying season or in an environment where there is a dearth of water. The antiproliferative test carried out using the reduction in the length of the radicles suggested the probable application of the plant material as herbicidal, allelopathic or antitumor agents. The plant extract may exhibit this by interfering with the biochemical system and/or other growth-related systems, like DNA division. The fact that the concentrations of the extract and the organic solvent fractions required to inhibit the growth of the guinea corn radicle lengths were higher than those that imparted lethality on the tadpoles implied the variations in the physiological compositions of the two organisms and hence the observed differences in the activities.

Like other plants in the compositae (Asteraceae) family, *S. sparganophora* produces many seeds, and this, in some cases, may account for the dominant nature of the plants in the family on farmlands or in the wild. It is possible that this property is accompanied by the secretion of certain chemical constituents into the surrounding soil, which may deter the germination and subsequent growth of other plants. Among the phytochemical constituents reported to be abundant in the family are the sesquiterpene lactones, polyacteylenes, alkaloids, monoterpenes and various phenolic derivatives.[12] The aqueous fraction of the plant was observed to be more active on both the tadpole lethality and inhibition to the growth of guinea corn radicles. On the tadpoles, this assertion was indicated by the LC50 of 5 μg/ml obtained for the aqueous fraction over 6.25 and 24 μg/ml obtained for the chloroform fraction and crude extract, respectively. Also, while the methanol extract at 4 mg/ml reduced the radicle length to 47.16% compared with the control, the aqueous fraction reduced the growth of the radicles to 43.81%. These suggested the probable presence of the major constituents responsible for these activities in the aqueous fraction and also that their presence in a more concentrated form may be responsible for the low LC50 (5 μg/ml) the fraction showed over both the crude extract and the chloroform fraction.

The biological activities of the medicinal plant are direct reflections of the effect and nature of the phytochemicals it contains. *S. sparganophora* has been observed to contain protocatechuic acid, p-coumaric acid and caffeic acid.[13] Because of their relative polarities, indicated by their phenolic compositions, these compounds are likely to be found in the aqueous fraction of the plant. Protocatechuic acid has been reported to be one of the allelopathic constituents of *Delonix regia*.[14] Also, in experimental animals, the compound has been reported to prevent chemically induced carcinogenesis.[15] The probable presence of this compound may have contributed to the biological activities of *S. sparganophora*.

Certain medicinal plants in the Asteraceae (Compositae) family have been reported to possess related activities. The allelopathic effects of Ambrosia cumanensis H.B.K. (Compositae) and the leaves and roots of Piqueria trinervia (Compositae) have been reported in the literature.[16,17] Also, the antiproliferative effects of some Asteraceae (Compositae) species against human cancer cell lines have been observed.[10] Furthermore, the cytotoxic effects of *Sonchus oleraceus* and one of its constituents, loliolide, against mice, rat and human cell lines have been established.[18] In the same way, the anticancer effects of the leaves of *Ageratum conyzoides* L. (Compositae) have been established scientifically.[19] Although this investigation requires further tests using appropriate human cell lines, the results obtained so far have indicated the claimed ethnomedicinal use of these plants in treating tumor-related ailments.
